# Gender differences in responses to an altruistic message regarding rubella vaccination

**DOI:** 10.3389/fpubh.2024.1353091

**Published:** 2024-08-09

**Authors:** Hideo Okuno, Hiroshi Satoh, Satoru Arai, Motoi Suzuki, Toshiko Kikkawa

**Affiliations:** ^1^Center for Surveillance, Immunization, and Epidemiologic Research, National Institute of Infectious Diseases, Tokyo, Japan; ^2^Department of Pediatric Emergency Medicine, Osaka City General Hospital, Osaka, Japan; ^3^Environmental Health Division, Takasaki City Health Center, Gunma, Japan; ^4^Faculty of Business and Commerce, Keio University, Tokyo, Japan

**Keywords:** risk communication, vaccine hesitancy, altruism, gender difference, rubella vaccination, group-interest, self-interest

## Abstract

**Background:**

The supplementary immunization activity (SIA) for the rubella vaccination of adult men born between 1962 and 1978 began in 2019 in Japan because of a vaccine gap in the cohort, as vaccination was not mandatory for those born in that period. However, SIA coverage remains low, despite an active campaign and financial support.

**Methods:**

We conducted a randomized controlled study based on a 2 (scenario: self-vaccination, child vaccination) × 2 (message: self-interest, group-interest) factorial design, using a Japanese online panel. Participants with children were assigned to the child vaccination scenario in Intervention 1, whereas others were assigned to the self-vaccination scenario. After Intervention 1, all participants were given the same information about rubella. In Intervention 2, participants assigned to self-interest messages received a message emphasizing the risk of rubella, and those assigned to group-interest messages received a message emphasizing herd immunity. After Intervention 2, we evaluated the effects using a questionnaire.

**Results:**

Among the 2,206 participants, information regarding rubella was evaluated as more reliable in the group—than in the self-interest message condition, especially among women. Women evaluated the necessity of rubella vaccination for adult men and women to be higher in the child-vaccination scenario and group-interest messages. However, no differences were found among men. By contrast, men exposed to the self-interest message positively evaluated the reliability of the rubella explanation.

**Conclusion:**

The findings indicate that emphasizing self-interest messages is more effective for men in promoting herd immunity against rubella.

## 1 Introduction

Although rubella is usually a mild, febrile illness and up to 50% of rubella infections are asymptomatic, congenital rubella syndrome (CRS) can occur in the developing fetuses of pregnant women infected with the rubella virus in early pregnancy. In Japan, a rubella outbreak occurred between early 2012 and late 2013, particularly among adult men (1, 2). Studies have indicated that this outbreak reflects fluctuating, yet incomplete, immunization policies for rubella in Japan. The initial Japanese rubella vaccination policy was introduced only for junior high school girls born between 1962 and 1978, to prevent CRS. Boys born in the same period did not have the opportunity to be vaccinated, creating a vaccine cohort gap when they became adults (3). Another outbreak occurred in mid-2018 in the Tokyo metropolitan area and other large cities. Seroepidemiological surveys among Japanese residents indicated that among men, the pocket susceptible to rubella virus, those 30–50 years, as determined in 2013, remained unchanged in 2018 (2, 4). One survey indicated that promoting revaccination among adult men to minimize the susceptible pocket is important for creating herd immunity and preventing rubella and CRS outbreaks (5). Therefore, the Japanese government started a supplementary immunization activity (SIA) for men born between 1962 and 1978, with the vaccination fee sponsored by the government (3, 6).

In addition to the nationwide campaign, some local governments conducted additional campaigns. For example, Kawasaki City added a catch-up vaccination campaign targeted at planning pregnant women and their partners, in addition to the vulnerable cohort of men in the middle of the outbreak. It revealed early and intensive vaccination and earlier decay of the outbreak (7). The campaign of Chiba City focused on the women who were planning to get pregnant and reported a decrease in infection (8). The city recommended women and families to visit clinics to receive antibody tests for free. It used the city magazine and website as the media for the campaign.

While some additional local government campaign efforts were conducted, the whole SIA coverage remains low despite active campaigns and financial support. Although the number of men eligible for rubella vaccination was estimated at approximately 15 million as of April 1, 2019, only 2.4% had been vaccinated by May 2020 (3). The reasons for the low coverage of the SIA program could be two fold (i) campaign messages may not have been effective in motivating the target group and (ii) behavioral costs for the target men may have been high despite having the motivation to be vaccinated, as vaccination requires two visits to clinics in the SIA program to receive an antibody test and subsequent vaccination. For full-time workers, this two-step procedure could be time-consuming and may decrease their motivation to be vaccinated (9).

This study focused on the first reason, which is the effectiveness of campaign messages. Motivating men to get vaccinated entails difficulty in conveying the significance of vaccination, as there seems to be no direct benefit for the target population since they are ordinary people with little knowledge about the occurrence of encephalitis or arthritis when infected. Furthermore, this strategy is weak because it seems to rely only on altruism and voluntary behavior.

In this study, we explored how the rubella vaccination rate can be increased for adult men. First, we explained the importance of this issue in the context of Japan. Next, we introduced various efforts by the government to increase the vaccination rate, e.g., policy changes and campaigns. Then, we evaluated that these efforts have not been effective thus far, i.e., they have not been contributing to an increase in the vaccination rate, thereby demonstrating that the efforts are flawed. Here, we focused specifically on the campaign’s message that emphasized the target population’s (adult men) altruism and the complicated process of vaccination even when vaccines are free of charge. In this regard, the effectiveness of utilizing altruism to increase the willingness to be vaccinated has been established in many studies, and the same method was utilized in the governmental campaign (10–12). However, considering the campaign’s ineffectiveness, we examined the message content, which appealed to self-interest, since some recent studies have examined this factor. Finally, we clarified that if self-interest would affect the decision to be vaccinated, gender differences could be worth examining since women have greater concerns about vaccination than men (13).

In addition, considering the current target demographic of adult men, some studies, such as those by Elawad et al. in 2017 and Gualano et al., in 2022, have suggested more effective campaign methods that have proven to be encouraging or that offer opportunities for vaccination in the workplace (14, 15). However, the Japanese government cannot use workplaces or allow mass vaccination because of legal restrictions. In principle, adult vaccination occurs voluntarily. The only exception was the COVID-19 vaccination in the summer of 2021, which was conducted in workplaces and involved group vaccination. However, this policy was introduced only in a state of urgency, when COVID-19 cases experienced a surge and the Olympic Games were to be held in Japan at the same time.

In addition to rubella, the number of studies focusing on altruistic motivations for vaccination has increased. Studies have shown that vaccination intention generally increases for COVID-19, influenza, and other vaccines when a message induces altruistic motivation. For instance, altruism-eliciting videos increased younger adults’ intentions to be vaccinated against COVID-19 (10). The importance of altruistic behavior for COVID-19 vaccinations is similar to that for influenza, as younger people are the major transmitters, whereas the victims of influenza-related deaths are people aged over 65 years (11). In contrast, the motivation to receive COVID-19 and influenza vaccinations may not rely merely on altruism or an understanding of herd immunity, as these diseases can affect anyone and may evoke self-interest motivation. A previous study examined self-interest in the decision to be vaccinated (16). However, the study showed that people inferred that others were more motivated by self-interest and did not focus on the effects of self-interest messaging.

In this study, we focused on gender differences, i.e., differences between women and men, because the involvement of women in the rubella infection issue is more than that of men. Specifically, if women are infected by unvaccinated men, their children may develop the disease (CRS) after birth. Gender differences were also reflected in the governmental policy regarding the initial Japanese rubella vaccination, which was introduced only for junior high school girls and did not include junior high school boys. Previous studies have examined gender differences in occupational hazards from the perspective of vulnerability to health risks (17). Our study did not focus on biological differences. Rather, we emphasized the difference of interests between genders.

The biggest obstacle to rubella vaccination among adult men in Japan is the lack of self-concern and recognition of the possibility of being affected by rubella infection (13). Therefore, another rationale for motivating target men, aside from altruism, should be provided, such as explaining the benefits of vaccination. The primary benefit of vaccination is preventing the adverse effects of rubella infection. However, many adults do not recognize the consequences of the infection or the benefits of vaccination, as rubella is believed to be a disease that mainly affects children in Japan. Therefore, information on the negative consequences of rubella infection in adult men may motivate them to be vaccinated.

To examine this inference, we conducted an online experiment to determine the effects of two types of messages: one emphasizing self-interest and the other emphasizing herd immunity (hereafter, group-interest). We predicted that a self-interest message would be more effective than a group-interest message.

Additionally, two fictitious scenarios were used to examine whether there was a difference between high and low self-relevance. If the vaccination was for oneself (self-vaccination scenario), participants might be more willing to be vaccinated than if the vaccination was for children (child-vaccination scenario). The difference between the two produces could induce differences between genders, as women tend to have higher personal relevance due to the possibility of pregnancy and from the potential for their child to be affected by CRS.

## 2 Materials and methods

### 2.1 Study design

A randomized controlled study based on a 2 (Intervention 1, vaccination scenario: self-vaccination vs. child vaccination) × 2 (Intervention 2, message: self-interest vs. group-interest) factorial design was conducted in January 2019. The study participants were recruited through an online panel provided by the NTTCom Online Marketing Solutions Corporation in Japan. This panel, which included 21,700,000 members as of June 2017, is one of the largest Internet panels in Japan (Tokyo, Japan). The company sent recruitment emails to all the members. Among them, those in their 20s and 50s gave informed consent online when they started participating in the survey.

### 2.2 Procedure

This study obtained ethical approval from the institutional ethics committee of the National Institute of Infectious Diseases of Japan (Approval number: 962).

A total of 2,206 participants comprising approximately equal percentages of gender and age groups (20–50s) were recruited. The mean age of the participants was 45.4 years (SD, 8.2) (Table 1). Approximately two-thirds of the participants (*n* = 1,548, 70%), were married and approximately half had children aged 15 years and younger (*n* = 1,046, 47%). Furthermore, among them, more men than women had graduated from graduate school or university (Table 1).

**Table 1:** Participant characteristics.

		All participants		Men		Women	
		*n* = 2,206		*n* = 1,090	(%)	*n* = 1,116	(%)
Mean age (years, ±SD)		45.4 (±8.2)		47.0 (±7.7)		43.8 (±8.3)	
Marital status							
	Married	1,548	70	728	67	820	73
	Unmarried	535	24	308	28	227	20
	Divorced/Widowed	123	6	54	5	69	6
Having children <15 years		1,046	47	511	47	535	48
Education							
	High school/Junior college	1,015	46	368	34	647	58
	University	1,017	46	597	55	420	38
	Graduate school	150	7	111	10	39	3

The experiment involved two phases: (a) presenting vaccination scenarios (Intervention 1) and (b) presenting messages (Intervention 2) to the participants. In Intervention 1, participants were assigned to the vaccination scenario depending on their status of parenthood, and in Intervention 2, they were randomly assigned.

The participants first visited an independent portal page and were asked to provide informed consent if they chose to participate. After obtaining informed consent, they were asked whether they had children aged 15 years or younger. Those who answered that they had children were assigned to the child-vaccination scenario in Intervention 1, and the remaining were assigned to the self-vaccination scenario. At this stage, participants were controlled depending on their parenthood status. In Intervention 2, participants were randomly assigned to one of the two experimental conditions (self- or group-interest message), irrespective of the assignment in Intervention 1. After Intervention 2, all participants completed a questionnaire that evaluated the reliability and intelligibility of the rubella explanation, anxiety over vaccine side effects, and the necessity of rubella vaccination for adult men and women (Supplementary Figure 1). Responses were rated on a five-point Likert-type scale ranging from 1 (not at all) to 5 (extremely).

### 2.3 Intervention 1: vaccination scenario

Intervention 1 included two scenarios: self-vaccination and child vaccination. The difference between the two conditions was in the statements provided to the participants. Participants assigned to the self-vaccination scenario were told the following: “Imagine a situation in which you had received an explanation of rubella and the rubella vaccination from a doctor before receiving the rubella vaccine.” This was followed by a general explanation. Then, participants of both conditions were asked to read the same explanation about rubella and rubella vaccines (see Supplementary Figure 2). The introduction provided general information on rubella and rubella vaccines. All information was adapted from the published content on the official website of the National Institute of Infectious Disease (NIID). The participants in the child-vaccination scenario were told the following: “Imagine a situation in which you had received an explanation of rubella and the rubella vaccination from a doctor before your child received the rubella vaccine.” Then, the same general explanation followed (Supplementary Figure 2).

### 2.4 Intervention 2: message

Intervention 2 involved two message conditions: self-interest and group-interest. In the self-interest message condition, participants were presented with the following information regarding rare but severe complications caused by rubella: “Reportedly, encephalitis occurs in approximately one per 4,000–6,000 cases, pancytopenia occurs in approximately one per 4,000–6,000 cases, and arthritis occurs in 5–30 per 100 cases, which would result in hospitalization.” This information emphasized the negative impact of rubella infection. We inferred that the information provided would elicit self-interest in rubella vaccination among participants.

Conversely, in the group-interest message condition, participants received the following information: “If not only women of childbearing age and children but many other people receive rubella vaccines, the number of people who are infected as a whole can be reduced. Not only vaccinated people but also those who cannot receive the vaccine, such as pregnant women and people with weakened immunity, can be protected.” This information emphasized the significance of rubella vaccination in achieving herd immunity.

### 2.5 Measurement

We analyzed the data to evaluate the impact of the vaccination scenarios and messages and compared the responses of participants based on their gender. The differences among groups were analyzed by performing a two-way factorial ANOVA, with *p* < 0.05 considered statistically significant. All analyses were conducted using IBM SPSS ver.27.

## 3 Results

### 3.1 Reliability and intelligibility of rubella explanation and anxiety regarding vaccine side effects

**Figure 1:**
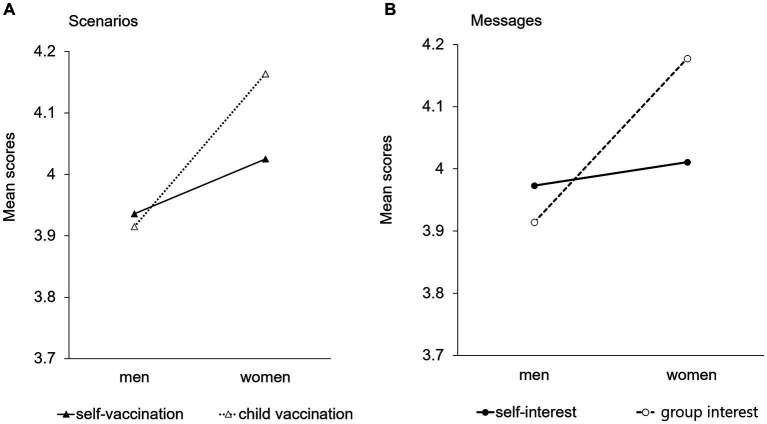
Gender differences in the reliability evaluation of rubella explanations between two scenarios and messages. **(A)** Scenarios. **(B)** Messages.

Regarding the reliability of the rubella explanation, the ANOVA results revealed a significant interaction between gender and scenario (*p* = 0.03, Figure 1). These results indicate that the difference in the scenario did not yield a significant difference for men, whereas reliability was evaluated as higher for the child than for the self-vaccination scenario among women. Additionally, a significant interaction between gender and messages was observed (*p* < 0.01, Figure 1). Self-interest messages yielded no difference between men and women, whereas group-interest messages were evaluated as more reliable among women than among men.

Similarly, regarding the intelligibility of the rubella explanation, the results revealed significant interactions between gender and scenario (*p* < 0.01, Table 2) and between gender and message (*p* = 0.04, Table 3, Supplementary Figure 3). However, regarding anxiety regarding vaccine side effects, a three-way ANOVA did not reveal any significant main effects or interactions (Tables 2, 3).

**Table 2:** Mean scores on the Likert-type scales of the self- and child-vaccination scenarios among men and women.

		Participants				Gender × vaccine scenarios		
		Men		Women				
		*n* = 1,090	(95% CI)	*n* = 1,116	(95% CI)	df	*F* value	*p* value
**Willingness to be vaccinated**								
Self-vaccination scenario		3.202	3.109–3.295	3.208	3.116–3.300	1	9.663	0.002^a^
Child-vaccination scenario		3.494	3.393–3.594	3.802	3.706–3.899			
**Reliability of rubella explanation**								
Self-vaccination scenario		3.936	3.866–4.005	4.025	3.956–4.094	1	4.772	0.029^a^
Child-vaccination scenario		3.915	3.840–3.991	4.164	4.092–4.236			
**Intelligibility of rubella explanation**								
Self-vaccination scenario		3.914	3.842–3.987	4.013	3.941–4.085	1	7.859	0.005^a^
Child-vaccination scenario		3.887	3.808–3.966	4.200	4.124–4.275			
**Anxiety regarding vaccine side effects**								
Self-vaccination scenario		3.166	3.079–3.252	3.273	3.187–3.358	1	3.181	0.075
Child-vaccination scenario		3.314	3.221–3.408	3.260	3.170–3.346			
**Necessity of rubella vaccination for adult women**								
Self-vaccination scenario		4.04	3.963–4.118	4.111	4.034–4.188	1	16.076	<0.001^a^
Child-vaccination scenario		3.997	3.913–4.082	4.395	4.314–4.475			
**Necessity of rubella vaccination for adult men**								
Self-vaccination scenario		3.816	3.736–3.896	4.024	3.944–4.103	1	13.87	<0.001^a^
Child-vaccination scenario		3.824	3.737–3.911	4.346	4.263–4.429			

^a^ANOVA revealed a significant interaction between the impact of gender and the vaccine scenarios.

**Table 3:** Mean scores on Likert-type scales of the self- and group-interest messages among men and women.

		Participants				Gender × messages		
		Men		Women				
		*n* = 1,090	(95% CI)	*n* = 1,116	(95% CI)	df	*F* value	*p* value
**Willingness to be vaccinated**								
Self-interest message		3.355	3.261–3.450	3.456	3.363–3.550	1	1.331	0.249
Group-interest message		3.34	3.241–3.439	3.554	3.459–3.648			
**Reliability of rubella explanation**								
Self-interest message		3.937	3.866–4.008	4.011	3.940–4.081	1	6.779	0.009^a^
Group-interest message		3.914	3.840–3.989	4.178	4.107–4.249			
**Intelligibility of rubella explanation**								
Self-interest message		3.904	3.830–3.978	4.034	3.961–4.108	1	3.886	0.049^a^
Group-interest message		3.897	3.820–3.975	4.179	4.104–4.253			
**Anxiety regarding vaccine side effects**								
Self-interest message		3.289	3.201–3.377	3.274	3.187–3.362	1	0.82	0.365
Group-interest message		3.191	3.099–3.283	3.258	3.170–3.346			
**Necessity of rubella vaccination for adult women**								
Self-interest message		4.044	3.965–4.124	4.201	4.122–4.279	1	3.635	0.057
Group-interest message		3.993	3.910–4.076	4.305	4.226–4.384			
**Necessity of rubella vaccination for adult men**								
Self-interest message		3.821	3.739–3.903	4.101	4.020–4.182	1	4.066	0.044^a^
Group-interest message		3.819	3.733–3.904	4.269	4.187–4.350			

^a^ANOVA revealed a significant interaction between the impact of gender and the messages.

### 3.2 Evaluation of the necessity of rubella vaccination for adult men and women

**Figure 2:**
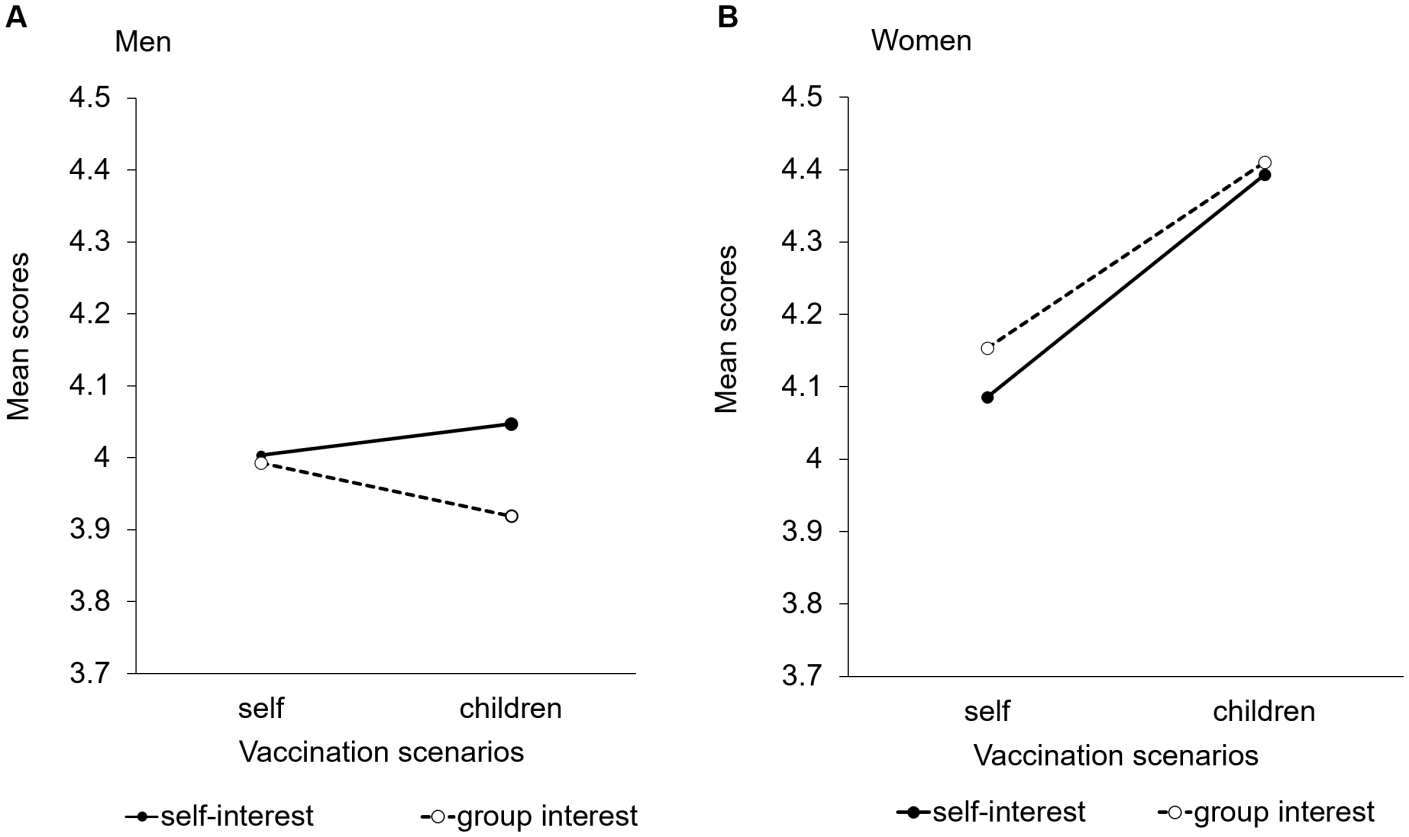
Evaluation of the necessity of rubella vaccination for adult women among men and women. **(A)** Men. **(B)** Women.

Women rated the necessity of rubella vaccination for adult women higher than men (Figure 2). No significant interactions or main effects between the scenarios and messages were found among men. ANOVA revealed a significant main effect for the scenarios (*p* < 0.01) among women. The average score for the child-vaccination scenario was higher than that for the self-vaccination scenario (Figure 2).

**Figure 3:**
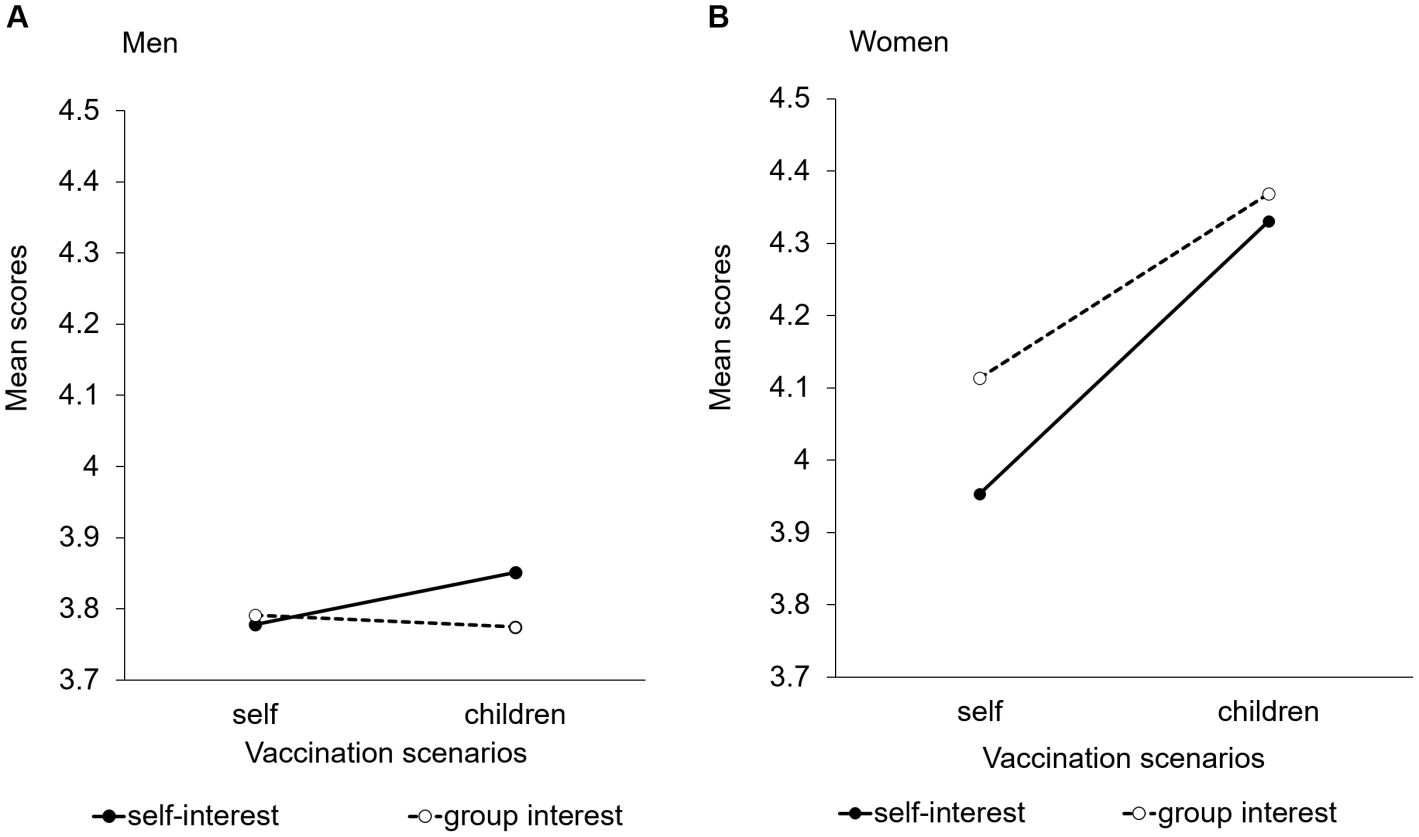
Evaluation of the necessity of rubella vaccination for adult women among men and women. **(A)** Men. **(B)** Women.

No significant interactions or main effects were detected between the scenarios and messages among men (Figure 3) regarding the evaluation of the necessity of rubella vaccination. In other words, men did not feel the necessity of vaccination irrespective of the experimental conditions. In contrast, women rated the necessity of rubella vaccination for adult men higher than men (Figure 3). A two-way ANOVA with scenarios and messages revealed a significant main effect for scenarios (*p* < 0.01) and messages (*p* < 0.05) among women. The average score in the child-vaccination scenario was higher than that in the self-vaccination scenario, regardless of the differences in the messages (Figure 3).

## 4 Discussion

In this study, we examined how the rubella vaccination rate can be increased for adult man. The results showed that the group-interest message and the child-vaccination scenarios were evaluated more positively by women. This suggests that in clinical settings, when explaining vaccination for children to mothers, clinicians should present not only self-but also group-interest messages for better risk communication, as these messages could be more effective than self-interest messages for mothers. However, among men, group-interest messages were not as effective as self-interest messages.

Indirect support exists for this discussion from a cultural perspective. According to the 2021 Charities Aid Foundation (CAF) index, the Japanese are least willing to help others (18). This result partially supports that we cannot rely on a campaign that appeals to people’s altruism, e.g., “Being vaccinated yourself prevents children whom you may not know from illness.” The complex two-step vaccination procedure initiative of the Japanese government may have increased the unwillingness to help others among Japanese men who were the target of the campaign.

These results suggest that self-interest messages may effectively promote herd immunity. The reliance on people’s altruistic motivation has attracted significant research interest, and evidence for this has accumulated, especially since the COVID-19 pandemic (19, 20). We recommend emphasizing individual advantages, even if herd immunity is important, as well as prosocial behaviors, to promote the advantages of vaccination and increase the motivation to be vaccinated.

However, the importance of altruism cannot be denied when considering public health campaigns since many studies have established it to be a strong facilitator for establishing herd immunity (12). In addition, Tarimo et al. (21) reported on gender differences, i.e., women were more motivated by altruism than men. However, based on our results and the Japanese government’s failure in the rubella campaign, relying on altruism alone to establish public health might be a risky strategy. If public health experts have various perspectives or alternative methods of conveying messages other than altruism, they can adopt more flexible strategies for changing people’s behaviors. Self-interest is one such strategy. However, research in this area thus far is scant (14), so we aim to accumulate further studies and investigate other additional factors that may motivate people toward vaccination and contribute to public health. Vaccination is a typical area requiring people’s understanding and cooperation, without which situations similar to what was experienced during COVID-19 might occur.

This study has some limitations. First, data were collected using a Japanese online panel. Japanese society has unique characteristics such as high masculinity and power distance (22). Moreover, previous studies have noted that message effects differ across cultures (23). Thus, further studies are needed to confirm whether our results can be generalized to other countries and cultures.

Second, although this study focused on the rubella vaccine, a previous study of the influenza vaccine indicated that altruistic behaviors are associated with inoculation behaviors, even in Japan (24). Moreover, the self-interest message regarding the human papillomavirus (HPV) vaccine could be more effective for women, as cervical carcinoma associated with HPV infection is a more realistic threat to women than to men. In some countries, men are eligible for HPV vaccines, as the effectiveness of HPV vaccines in men has been reported (25). A previous study indicated that altruistic motives are important for vaccine acceptance among men (26), indicating that the responses of participants could change depending on gender.

Finally, previous studies have focused mainly on altruistic behaviors or motivational aspects when herd immunity is crucial for protecting society. Consequently, little attention has been paid to the importance of self-interest messages in motivating men to receive the rubella vaccine in Japan, as demonstrated by the failure of the Japanese governmental campaign that focused on altruism. In addition, the focus on the self-interest factor sheds light on the strategies of previous vaccine campaigns for the general public who have different values, interests, and so on, which Perloff points out their limitations by reviewing various campaign practices (27). If we consider this motivation and include it in future studies, we can gain more insight into vaccination strategies, especially from a risk communication perspective.

## 5 Conclusion

This study demonstrated that women evaluated group-interest messages and child-vaccination scenarios more positively than men. It also demonstrated that men did not positively evaluate the group-interest messages regarding rubella vaccines. This indicates that emphasizing self-interest messages is more effective for men in increasing their herd immunity to rubella.

## Data availability statement

The original contributions presented in the study are included in the article/Supplementary material; further inquiries can be directed to the corresponding author.

## Ethics statement

The studies involving humans were approved by National Institute of Infectious Diseases. The studies were conducted in accordance with the local legislation and institutional requirements. The participants provided their informed consent to participate in this study.

## Author contributions

HO: Writing – original draft. HS: Conceptualization, Supervision, Writing – review & editing. SA: Conceptualization, Supervision, Writing – review & editing. MS: Funding acquisition, Supervision, Writing – review & editing. TK: Conceptualization, Funding acquisition, Writing – original draft.
