# Supplementary material for: Gender differences in responses to an altruistic message regarding rubella vaccination
Source: Front Public Health. 2024 Aug 9;12:1353091. doi: 10.3389/fpubh.2024.1353091 (PMC11341465; doi:10.3389/fpubh.2024.1353091)
Supplement: Supplementary file 1 [file Data_Sheet_1.PDF]

Rubella is an infectious disease caused by the rubella virus. Symptoms of rubella are characterized by fever, rash, and lymphadenopathy. Symptoms are often mild, and up to 50% of infections may be subclinical or inapparent. However, if a pregnant woman is infected with the rubella virus up to around 20 weeks of pregnancy, the baby will be born with defects in the eyes, ears, and heart, i.e., congenital rubella syndrome (CRS).

The rubella vaccine is effective in preventing rubella. For example, in the Americas, vaccination has significantly reduced the number of reported cases of rubella, and the elimination of rubella has been declared. However, rubella is still common in Asian countries, and there was an epidemic of rubella in Japan in 2012-2013. In Japan, an increase in rubella cases was also reported in 2018. Japanese government has decided to promote additional measures with the goal of eliminating rubella by 2020.

At least 95% of vaccinated children age 12 months or older show serologic evidence of rubella immunity after a single dose. Therefore, in Japan, all children aged ≥12 months are recommended to receive 2 doses of rubella vaccine. Due to the 2012-2013 rubella epidemic, 45 cases of congenital rubella syndrome were reported in Japan. However, there have been no reported cases of CRS babies born to mothers who had been vaccinated twice.

Supplementary Figure 2. Introduction message
