# Supplementary figures and images for: Gender differences in responses to an altruistic message regarding rubella vaccination
Source: Front Public Health. 2024 Aug 9;12:1353091. doi: 10.3389/fpubh.2024.1353091 (PMC11341465; doi:10.3389/fpubh.2024.1353091)

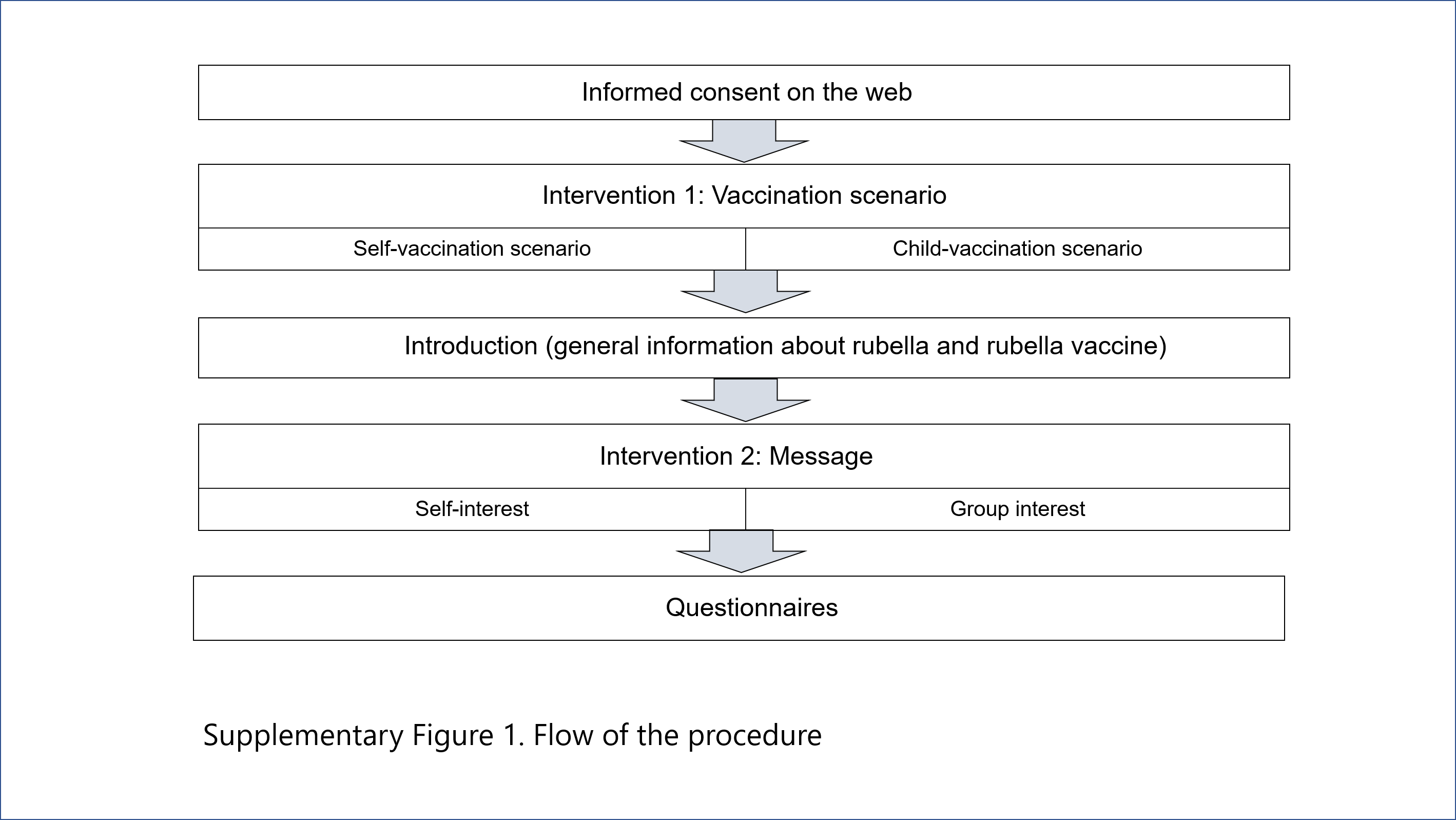

Supplement: Supplementary file 2 [file Image_1.TIF]

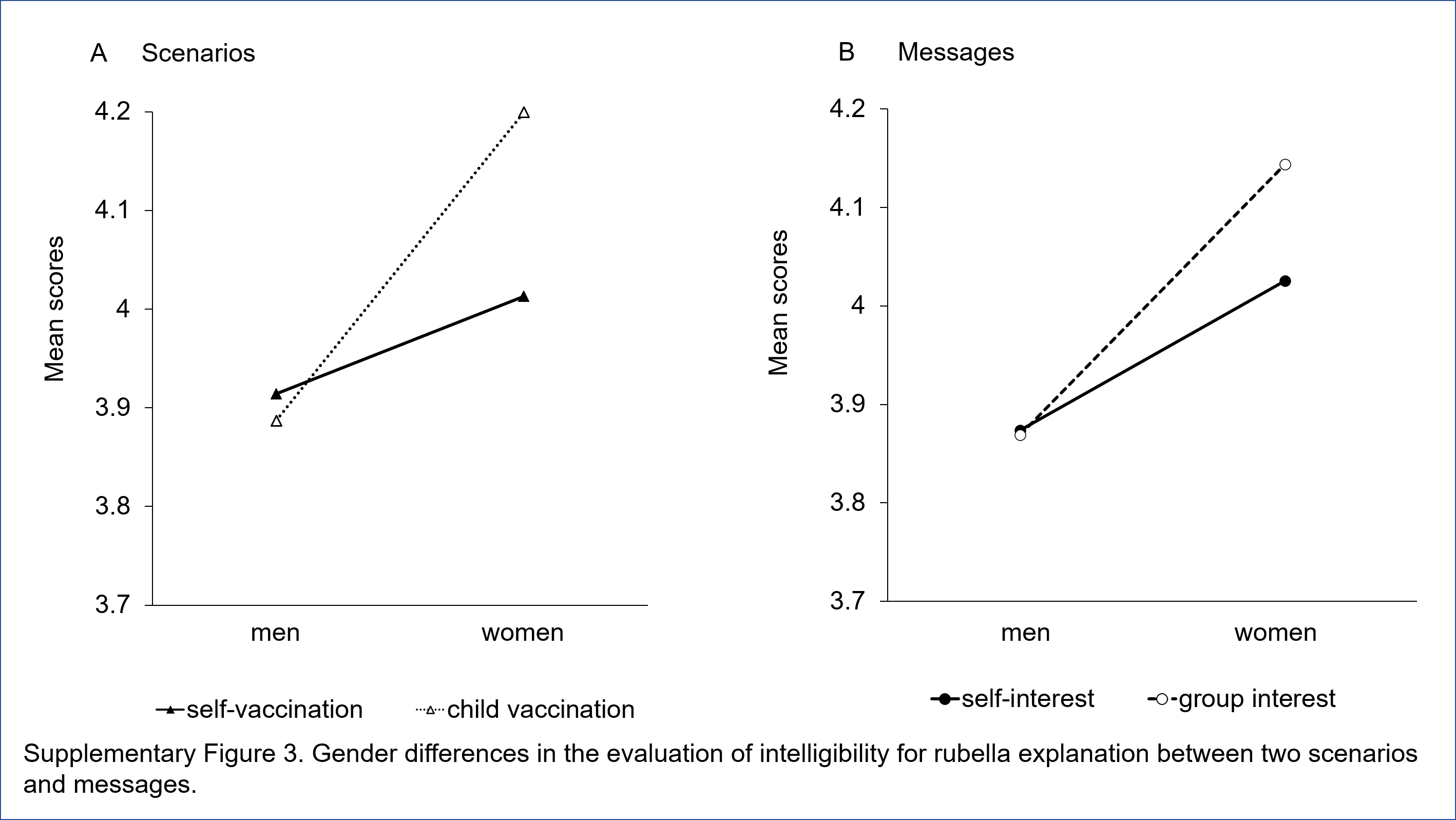

Supplement: Supplementary file 3 [file Image_2.TIF]
